# A Cooperative Phase-Steering Technique with On-Off Power Control for Spectrum Sharing-Based Wireless Sensor Networks

**DOI:** 10.3390/s20071942

**Published:** 2020-03-30

**Authors:** Sangku Lee, Janghyuk Yoon, Bang Chul Jung

**Affiliations:** Department of Electronics Engineering, Chungnam National University, Daejeon 34134, Korea; sklee@o.cnu.ac.kr (S.L.); jhyoon@o.cnu.ac.kr (J.Y.)

**Keywords:** cognitive radio networks, spectrum sharing, wireless sensor networks, cooperative communications, outage probability, cooperative phase steering

## Abstract

With the growth of the number of Internet of Things (IoT) devices, a wide range of wireless sensor networks (WSNs) will be deployed for various applications. In general, WSNs are constrained by limitations in spectrum and energy resources. In order to circumvent these technical challenges, we propose a novel cooperative phase-steering (CPS) technique with a simple on-off power control for generic spectrum sharing-based WSNs, which consists of a single secondary source (SS) node, multiple secondary relay (SR) nodes, a single secondary destination (SD) node, and multiple primary destination (PD) nodes. In the proposed technique, each SR node that succeeds in packet decoding from the SS and for which its interference power to the PD nodes is lower than a certain threshold is allowed to transmit the signal to the SD node. All SR nodes that are allowed to transmit signals to the SD node adjust the phase of their transmit signal such that the phase of received signals at the SD node from the SR nodes is aligned to a certain angle. Moreover, we mathematically analyze the outage probability of the proposed scheme. Our analytical and simulation results show that the proposed technique outperforms the conventional cooperative relaying schemes in terms of outage probability. Through extensive computer simulations, it is shown that the analytical results match well with the simulated outage probability as a lower bound.

## 1. Introduction

The Internet of Things (IoT) concept can be defined as all devices such as computing devices, mechanical and digital machines, objects, animals, or people in the world will be interconnected to collect and exchange data remotely for interaction with one another and with other devices and services over the Internet [1]. Due to the developments of less expensive small sensors, a ubiquitous network, efficient cloud computing, and big data, the IoT can be used in many application fields such as health care monitoring, environmental sensing, forest fire detection, natural disaster prevention, and battlefield surveillance [2]. Furthermore, it is projected that the worldwide number of IoT-connected devices will increase to 43 billion by 2023, an almost threefold increase from 2018 [3].

As part of an IoT topology, wireless sensor networks (WSNs) have been a key enabler of IoT, which can be described as a network of devices that can cooperatively sense the environment and may control the devices through a wireless link to communicate between persons, computers, or the surrounding environment [4]. As the number of IoT-connected devices increases, the demand for WSN data traffic will explosively increase, and the corresponding spectrum resources are needed to support such demand. However, WSN applications have been proposed to utilize the shared spectrum of the Industrial, Scientific, and Medical (ISM) band, the unlicensed operators of which can be permitted to use these bands. The shared ISM bands have become increasingly crowded, and the performance of the WSNs’ communication can be degraded due to the lack of spectrum bands [5]. However, discovering and supplying new spectrum bands is very challenging for most countries. If we want to exploit new spectrum bands, we have to develop a new communication technology with huge cost and effort. Meanwhile, it is found that most of the radio frequency spectrum bands are inefficiently utilized depending on time and place [6]. Hence, spectrum sharing techniques have received much attention from both industry and academia since they increase the utilization of the existing unused spectrum bands without changing the spectrum allocation policy [7,8,9]. In the spectrum sharing techniques, secondary/unlicensed users (SUs) are allowed to access primary users’ spectrum only if the transmission of primary/licensed users (PUs) is absent (i.e. interweave mode) or the quality-of-service (QoS) of the PUs is guaranteed (i.e. underlay mode). In interweave mode, SUs need to sense the spectrum band to check whether the spectrum is being occupied by PUs and to find the unused portion of the spectrum, and thus, spectrum sensing plays a significant role in increasing the spectrum efficiency [10,11]. Zhai et al. proposed the successive interference cancellation (SIC) technique-based cognitive decode-and-forward (DF) relaying scheme to mitigate the interference from primary transmitters [12]. In addition, novel communication techniques are presented such as a dynamic spectrum sharing technique in a multichannel IoT environment [13] and an optimal resource allocation technique with an NOMA-based clustered cooperative sensing environment in spectrum sharing-based industrial IoT networks [14].

WSNs are composed of nodes that typically are equipped with batteries having a restricted power supply. The deployment of sensor nodes usually occurs in inaccessible environments, and with the limited battery capacity, their transmit power should be constrained for their lifetime. Therefore, wireless sensor nodes usually communicate with one another at short distances, and relay networks are used for larger transmission distances. Furthermore, if the physical channel is bad, the power constraint of nodes in WSNs is the main factor that degrades the system performance of WSNs [15].

Therefore, data transmission with efficient energy consumption becomes an important design consideration for such networks. Meanwhile, cooperative communications with multiple relay nodes can collaborate to form virtual antenna arrays to achieve spatial diversity [16]. If WSNs exploit a cooperative communication system, they can achieve cooperative diversity among the nodes distributed in the network so as to achieve better energy efficiency by distributing the transmit energy consumption burden to multiple nodes [17,18]. A cooperative communication system can reduce the required transmission power under a certain throughput requirement due to spatial diversity. Hence, cooperative communication is one of the most important techniques to overcome the power limitation and improve the physical-layer performances of WSNs by providing additional spatial diversity with multiple relay nodes [19].

Among various cooperative relaying techniques, a selective decode-and-forward (SDF) protocol, also known as opportunistic relay selection (ORS), has been considered one of the most practical strategies in the spectrum sharing-based cooperative network [20,21]. However, the ORS scheme requires all the relays to transmit the pilot signal to the destination node and the feedback sequence of the destination node for the best relay node selection, which induces additional signal overhead on the network. Another cooperative relaying technique called cooperative phase steering (CPS) was proposed for cooperative relay networks. In the CPS scheme, relay nodes steer the phase of the transmit signals by pre-adjusting the phase distortion imposed by the communication channel to the destination node so that the phases of the received signals at the destination node are aligned. Moreover, since relay nodes in the CPS scheme only need local channel state information (CSI), CPS does not require the feedback information from the destination node so that it operates in a fully-distributed manner [22]. Meanwhile, to the best knowledge of the authors, conventional CPS has not been proposed for spectrum sharing environments.

Therefore, in this paper, as the main contributions, we modify the conventional CPS scheme for the spectrum sharing-based WSNs. Most importantly, only the secondary relay node that impacts the interference power less than certain interference constraints to the primary destination node can transmit the signal to the secondary destination node with the CPS scheme. By exploiting this method, we can strictly regulate interference to the primary destination node. Moreover, we mathematically analyze the outage probability of the proposed CPS technique in the spectrum sharing-based WSNs and demonstrate that the proposed CPS technique outperforms the conventional opportunistic relay selection scheme in terms of outage probability.

The rest of this paper is organized as follows. In Section 2, we briefly describe the system model considered in this paper. The overall procedure for the proposed CPS technique for spectrum sharing-based WSNs is explained in Section 3. In Section 4, The performance of the proposed CPS technique for spectrum sharing-based WSNs is mathematically analyzed in terms of outage probability. Computer simulation results on the outage probability and throughput of the proposed technique are shown in Section 5. Finally, conclusions are drawn in Section 6.

## 2. System Model

**Figure 1:**
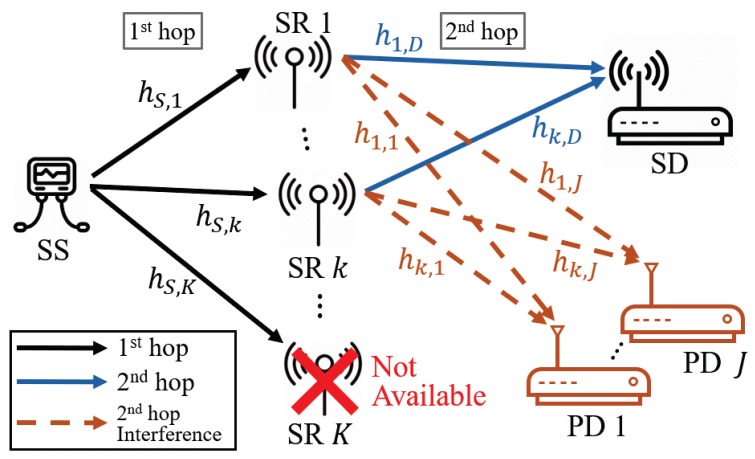
System model of a spectrum sharing-based mobile MANET. SR, secondary relay; SS, secondary source; SD, secondary destination; PD, primary destination.

We consider a spectrum sharing-based WSNs with multiple decode-and-forward (DF) relay nodes, as shown in Figure 1. There exist a single secondary source (SS) node, a single secondary destination (SD) node, *J* primary destination (PD) nodes, and *K* secondary relay (SR) nodes. The wireless communication channels between the SS node and the $k^{\mathrm{th}}$ SR node, between the $k^{\mathrm{th}}$ SR node and the SD node, and between the $k^{\mathrm{th}}$ SR node and the $j^{\mathrm{th}}$ PD node are defined as $h_{S,k}$, $h_{k,D}$, and $h_{k,j}$, respectively, as in Figure 1. Moreover, we assume that $h_{S,k}$, $h_{k,j}$, and $h_{k,D}$ follow an independent and identically distributed (i.i.d.) complex Gaussian distribution with zero mean and different variances, i.e., $h_{S,k}∼\mathrm{CN}\left(0,\sigma_{S,k}^{2}\right)$, $h_{k,j}∼\mathrm{CN}\left(0,\sigma_{k,j}^{2}\right)$, and $h_{k,D}∼\mathrm{CN}\left(0,\sigma_{k,D}^{2}\right)$. The variance of the wireless channels changes according to relative distance among communication nodes in the system model.

We assume that the transmitters of primary networks are located far enough from the secondary network so that they cannot cause any interference to the SR nodes and SD node. Furthermore, it is assumed that the SS node and SD node are far from each other and that the fading between them is severe because of various obstacles in the WSN. The SS node does not directly affect the SD node. Therefore, the SS node can communicate with the SD node only through SR nodes. In addition, we assume that each $k^{\mathrm{th}}$ SR node knows the channel state information (CSI) related to itself, which is called local CSI, e.g., $h_{S,k}$, $h_{k,j}$, and $h_{k,D}$. Note that because the system consists of multiple SR nodes, the local CSI assumption is a more efficient way than when the CSI is needed at the destination or source node because the signaling overhead can be reduced. In our paper, quasi-static frequency-flat fading is assumed, i.e., the wireless channel coefficients are constant during the two-hop transmission time and change independently for every two-hop transmission time.

## 3. CPS Technique for Spectrum Sharing-Based WSNs

In this section, we describe the overall procedure of the proposed CPS technique. In the first hop, the SS node sends the signal to all SR nodes, and the received signals at the $k^{\mathrm{th}}$ SR node are given by:

(1) $$\begin{array}{cc} y_{k} & =\;\sqrt{P_{S}}h_{S,k}x_{S}+n_{k} \end{array}$$

where $P_{S}$ and $x_{S}$ denote the transmit power and the transmitted signal of the SS node, respectively. $n_{k}$ represents additive complex Gaussian noises at the $k^{\mathrm{th}}$ SR node, and it is assumed to follow $\mathrm{CN}\left(0,1\right)$ without loss of generality.

In order to perform phase alignment in the second hop, all relay nodes must know the original signal without noise. Therefore, each $k^{\mathrm{th}}$ SR node attempts to decode the received packet $y_{k}$ at the end of the first hop, and we assume that the packet decoding succeeded if the received signal-to-noise ratio (SNR) is larger than a certain threshold. Then, we can define the index set of the SR nodes that succeed in the packet decoding as D:

(2) $$\begin{array}{c} D≜\left\{k\in K:P_{S}{\left|h_{S,k}\right|}^{2}\geq \rho_{th}\right\} \end{array}$$

where $\rho_{th}=2^{2R}-1$ and *R* indicates the required data rate when the SS node and the SD node directly communicate with each other. K represents the total set of SR nodes defined as {1,2,…,K}. Note that we assume two-hop communication between the SS node and the SD node, so that the required data rate for each hop needs to be double that of the direct communication system.

At the second hop, we define the interference power constraint, also known as the interference temperature, at each PD node as *Q*. The interference channel information between each SR node and PD nodes can be obtained at the SR node through the periodic sounding of pilot signals transmitted by PD nodes. Each SR node decides if its interference power from itself to the PD nodes is lower than the distributed interference constraint Q/K. Please note that the interference constraint is normalized with the total number of SRs, not the number of relays in the available set defined in (4), since additional signaling overhead is required to evaluate the number of available SRs. Furthermore, note that interference power constraint *Q* for each PD node can be automatically satisfied when each SR node satisfies its distributed interference constraint since the actual number of SR nodes that transmit the signal to the SD node is less than *K*. Then, the index set of the SR nodes that satisfy the interference power constraint at the second hop is defined as:

(3) $$\begin{array}{c} V≜\left\{k\in K:\max_{j\in J}\frac{P_{k}}{K}{\left|h_{k,j}\right|}^{2}<\frac{Q}{K}\right\}=\left\{k\in K:P_{k}\;\max_{j\in J}{\left|h_{k,j}\right|}^{2}<Q\right\} \end{array}$$

$P_{k}$ represents the transmit power of the $k^{\mathrm{th}}$ SR node. To normalize the total consumed power of SR nodes, $P_{k}$ is divided by *K*. Hence, we can define the available set of SR nodes that belong to both D and V as:

(4) $$\begin{array}{c} A≜\left\{k\;|\;k\in \left(D\cap V\right)\right\}. \end{array}$$

At the second hop, the SR nodes that belong to A steer the phase of the transmit signal so that the phases of all received signals at the SD node are aligned. Then, the transmit signal of the $k^{\mathrm{th}}$ SR node is given by:

(5) $$\begin{array}{c} x_{k}=\exp\left(-i∠h_{k,D}\right)x_{S}, \end{array}$$

where $∠h_{k,D}$ denotes the phase of $h_{k,D}$. Then, at the second hop, the received signal of the SD node is given by:

(6) $$\begin{array}{c} \;\;\;y_{D}=\;\;\sum_{k\in A}\frac{\;\;\sqrt{P_{k}}}{K}h_{k,D}x_{k}+n_{D}\;=\;\;\sum_{k\in A}\frac{\;\;\sqrt{P_{k}}}{K}\left|h_{k,D}\right|x_{S}+n_{D}, \end{array}$$

where $n_{D}$ denotes additive complex Gaussian noise at the SD node, which follows CN(0,1). Finally, the outage probability of the proposed CPS technique is given by:

(7) $$\begin{array}{c} P_{out}=\mathrm{Pr}\left\{{\left(\sum_{k\in A}\frac{\;\;\sqrt{P_{k}}}{K}\left|h_{k,D}\right|\right)}^{2}<\rho_{th}\right\}. \end{array}$$

## 4. Outage Probability Analysis of the CPS Technique for Spectrum Sharing-Based WSNs

In this section, we mathematically analyze the outage probability of the proposed CPS technique for the spectrum sharing-based WSNs. First, we should obtain the probability that each SR node successfully decodes and satisfies the interference power constraint in order to calculate the probability that the SR node belongs to the available set A. From (4), the successful decoding probability of each SR node is given by:

(8) $$P_{D}=\mathrm{Pr}\left\{|h_{S,k}{|}^{2}\geq R^{\prime }\right\}=\exp\left(-\frac{R^{\prime }}{\sigma_{S,k}^{2}}\right).$$

where $R^{\prime }$ denotes $\frac{2^{2R}-1}{P_{S}}$. Then, we can calculate the probability of satisfying the interference constraint by using the order statistics as follows:

(9) PI=Prmaxj∈J|hk,j|2≤Q′=∑m=1J(−1)mJm+1J−1m1−exp−(m+1)Q′σk,j2,

where $Q^{\prime }=\frac{Q}{P_{k}}$. Hence, the probability that SR nodes satisfy both conditions:

(10) PA=PD×PI=exp−R′σS,k2·∑m=1J(−1)mJm+1J−1m1−exp−(m+1)Q′σk,j2

Then, we can calculate the probability that *i* out of *K* SR nodes are available to transmit the signal as:

(11) PrA=i=KiPAi1−PAK−i

Now, we show the outage probability of the SD node, $P_{out}$, by exploiting the generally used outage probability expression in the cooperative relay system as follow:

(12) Pout=∑i=0KPr|A|=iPrE∣|A|=i=∑i=0KKiPAi1−PAK−iPr∑k∈Ahk,D≤R*∣|A|=i,

where $R^{*}=\sqrt{\frac{2^{2R}-1}{P_{k}}}$.

In (12), without loss of generality, $\mathrm{Pr}\left\{\sum_{k\in A}\left|h_{k,D}\right|\leq R^{*}|\left|A\right|=i\right\}$ is actually the same as $\mathrm{Pr}\left\{\sum_{k^{\prime }=1}^{i}\left|h_{k^{\prime },D}\right|\leq R^{*}|\left|A\right|=i\right\}$ because all channel coefficients are i.i.d. random variables (RVs). For simplicity, we define $\sum_{k^{\prime }=1}^{i}\;\left|h_{k^{\prime },D}\right|$ as RV $Z_{i}$. Solving this probability is equivalent to deriving a cumulative density function (CDF) of $Z_{i}$ for all *i*.

Since $\left|h_{k^{\prime },D}\right|$ follows a Rayleigh distribution, we first obtain the CDF of $Z_{i}$, which is a function of *i* RVs and can be represented by the sum of *i* independent RVs following the Rayleigh distribution. The probability density function (PDF) and the CDF of $Z_{1}$ are respectively known as follows:

(13) $$f_{Z_{1}}\left(z\right)=\frac{2z}{\sigma_{k,D}^{2}}\exp\left(-\frac{z^{2}}{\sigma_{k,D}^{2}}\right),\;F_{Z_{1}}\left(z\right)=1-\exp\left(-\frac{z^{2}}{\sigma_{k,D}^{2}}\right).$$

The exact PDF of RV $Z_{i}$ for i≥2 needs to be calculated with i−1 times convolution of $f_{Z_{1}}$ with itself. However, it is very complicated to operate more than two times convolution of $f_{Z_{1}}$ with itself even if we take any transform. Thus, we obtain the exact PDF of $f_{Z_{2}}$, which is the convolution of $f_{Z_{1}}$ with itself [23], and we approximate the actual distribution with a Gaussian distribution, which has same mean and variance of $f_{Z_{i}}$ when i≥3. The PDF and CDF of $Z_{2}$ are defined as follows:

(14) $$\begin{array}{c} \begin{array}{c} f_{Z_{2}}\left(z\right)=\frac{1}{\sigma_{k,D}}\sqrt{\frac{\pi }{2}}\left(\frac{z^{2}}{\sigma_{k,D}^{2}}-1\right)\exp\left(-\frac{z^{2}}{2\sigma_{k,D}^{2}}\right)\mathrm{erf}\left(\frac{z}{\sigma_{k,D}\sqrt{2}}\right)+\frac{z}{\sigma_{k,D}^{2}}\exp\left(-\frac{z^{2}}{\sigma_{k,D}^{2}}\right),\\ F_{Z_{2}}\left(z\right)=\int_{0}^{z}f_{Z_{2}}\left(t\right)dt=1-\exp\left(-\frac{z^{2}}{\sigma_{k,D}^{2}}\right)-\frac{z}{\sigma }\sqrt{\frac{\pi }{2}}\exp\left(-\frac{z^{2}}{2\sigma_{k,D}^{2}}\right)\mathrm{erf}\left(\frac{z}{\sigma_{k,D}\sqrt{2}}\right). \end{array} \end{array}$$

As mentioned earlier, the PDF of $f_{Z_{i}}$ for i≥3 is approximately derived as the Gaussian distribution, which has the same mean and variance by using the central limits theorem. Therefore, we firstly derive the mean and variance of $Z_{1}$. The mean and variance of $Z_{1}$ are denoted by $\mu_{z}$ and $\sigma_{z}^{2}$ as follows:

(15) $$\begin{array}{c} \mu_{z}≜E\left[Z_{1}\right]=\frac{\sigma_{k,D}\sqrt{\pi }}{2},\\ \sigma_{z}^{2}≜E\left[{\left(Z_{1}-\mu_{z}\right)}^{2}\right]=\left(\frac{4-\pi }{4}\right)\sigma_{k,D}^{2}. \end{array}$$

Since each $h_{k,D}$ is independent of the others, the mean and variance of $Z_{i}$ for i≥3 are $i\mu_{z}$ and $i\sigma_{z}^{2}$ as in [24]. Therefore, the approximated CDF of $Z_{i}$ for i≥3 can be written as follows:

(16) $$\begin{array}{c} F_{Z_{i}}\left(z\right)=\frac{1}{2}\left(1+\mathrm{erf}\left(\frac{z-i\mu_{z}}{\sqrt{2i\sigma_{z}^{2}}}\right)\right),\;i\geq 3. \end{array}$$

Finally, we obtain the outage probability of the CPS technique for spectrum sharing-based WSNs for all *K* as follows:

(17) Pout=∑i=0KPr{|A|=i}Pr∑k∈Ahk,D≤R*∣|A|=i=∑i=0KKi(PA)i1−PAK−iFZiR*.

where $F_{Z_{0}}\left(R^{*}\right)=1$. Furthermore, as another performance metric, the effective throughput of the CPS technique is defined as:

(18) $$\begin{array}{c} R_{\mathrm{eff}}=R\times \left(1-P_{out}\right). \end{array}$$

Since *R* is the required data rate of the entire system, it can be regarded as the transmit rate at which the SS node intended to transmit. Therefore, the effective throughput in (18) represents the average transmit rate when the SS node tries to transmit data with transmit rate *R*.

## 5. Numerical Results and Analysis

In this section, we compare the performance of the proposed CPS technique with the conventional opportunistic relay selection (ORS) [20,21] scheme in a spectrum sharing-based WSNs in terms of outage probability and throughput. The parameters in all simulations were set to be $\sigma_{S,k}^{2}=0\;\mathrm{dB}$, $\sigma_{k,D}^{2}=\sigma_{k,j}^{2}=-10\;\mathrm{dB}$; the required data rate of the entire communication was R=1bit/s/Hz; and other parameters are described in each figure.

**Figure 2:**
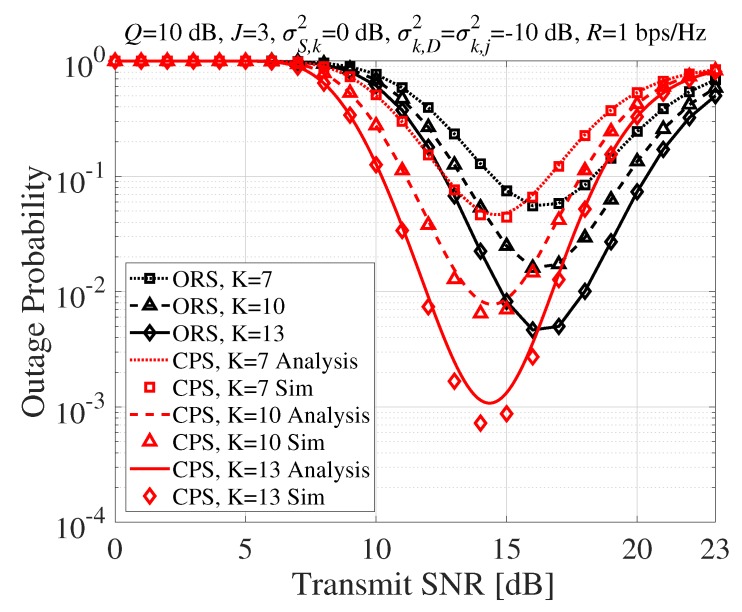
Outage probability according to the transmit SNR (dB). ORS, opportunistic relay selection; CPS, cooperative phase-steering; Sim, Simulation.

Figure 2 compares the outage probability performance of the proposed CPS with that of conventional ORS for varying the transmit SNR when the number of SR nodes is K=7,10,13. The proposed CPS technique provided higher performance than the ORS technique, which needed feedback for the index of a transmit SR node. We can see that the proposed CPS technique achieved the best performance at a lower transmit SNR than the conventional ORS technique, and its best performance was much better than the best performance of the ORS technique.

**Figure 3:**
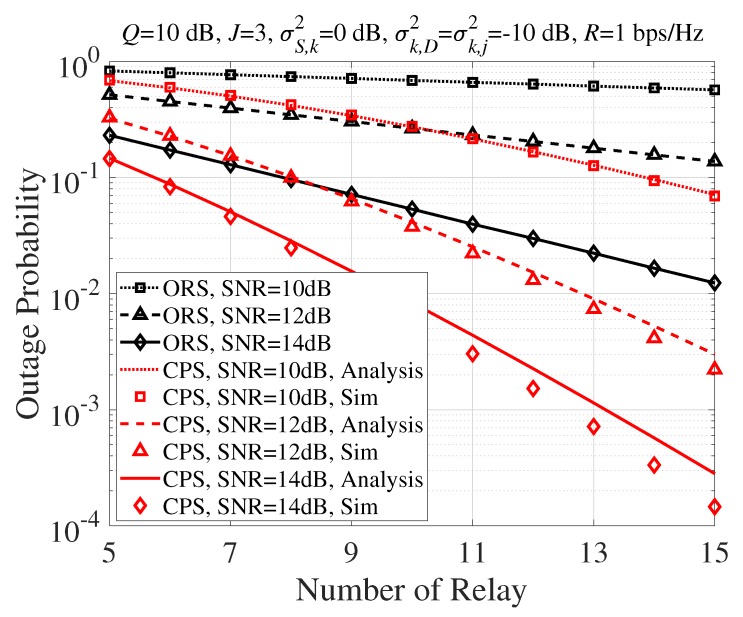
Outage probability according to *K*.

Figure 3 shows the outage probability performance with the number of SR nodes, *K*, when SNR=10,12,14 dB. The performance gap of the proposed CPS technique and ORS scheme was wider as *K* increased due to the increase of the spatial diversity gain. It is worth noting that the performance improvement of the proposed CPS technique became significant as the number of SR nodes increased.

**Figure 4:**
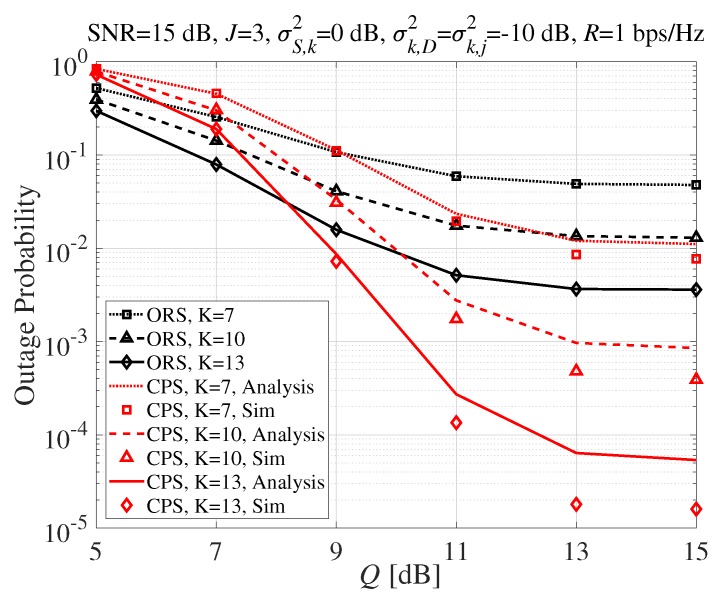
Outage probability according to *Q* (dB).

Figure 4 shows the outage probability performance of the proposed CPS technique according to the interference constraint, *Q*, when the transmit SNR=15dB with different numbers of SR nodes. It was observed that the outage probability of the proposed CPS technique outperformed the ORS scheme for high *Q* values. Furthermore, the performance improvement of the proposed CPS became significant as the number of SR nodes increased, as well, as described in Figure 3.

**Figure 5:**
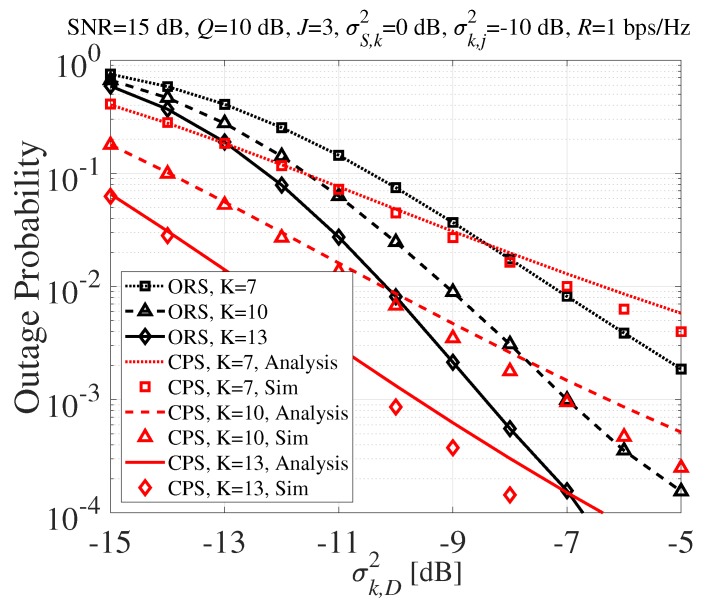
Outage probability according to $\sigma_{k,D}^{2}$ (dB).

The outage probability performance of the proposed scheme according to $\sigma_{k,D}^{2}$ is shown in Figure 5. A lesser amount of variance of the channel meant poor channel gain, which could be caused by deep fading, high path loss, long communication distance, etc. As we can see in Figure 5, the proposed CPS technique outperformed the conventional ORS technique when the channel was poor. This result showed that the diversity gain of many SRs with normalized power was a more efficient way to achieve better performance than the selection gain of one SR with integrated power when the channel was poor.

**Figure 6:**
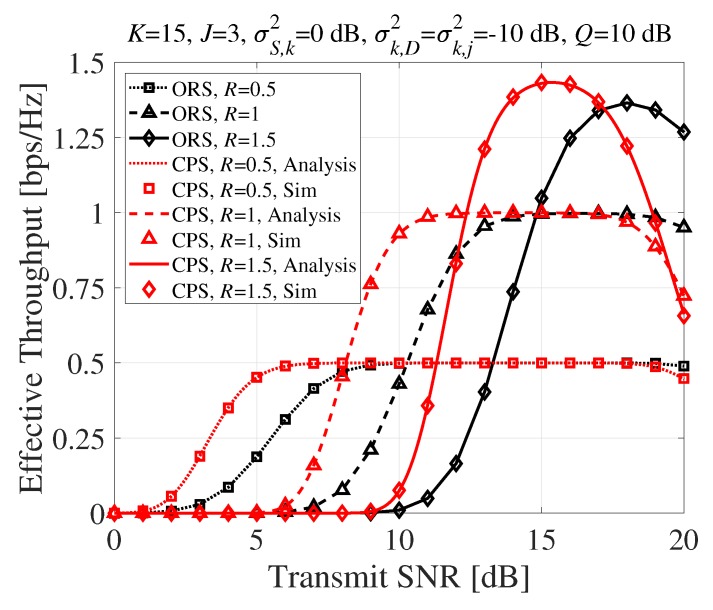
Effective throughput (bps/Hz) according to the transmit SNR (dB).

In Figure 6, the effective throughput performance was evaluated according to the transmit SNR for different required data rates. Because of the intended diversity gain imposed by phase alignment, the proposed CPS technique achieved maximum throughput with lower SNR than the conventional ORS technique. Moreover, the proposed CPS technique achieved slightly higher maximum effective throughput than ORS with lower SNR when R=1.5bps/Hz. However, even though the phase alignment was not intended to earn diversity gain at PDs, the summation of signals at PDs might have causes a small diversity gain. Because of that diversity gain, more SRs could be terminated more easily than ORS by not satisfying the interference constraint. Eventually, it was shown that the effective throughput of the CPS technique started to degrade at lower SNR when ORS started to degrade. Even so, it is worth noting that CPS could achieve maximum throughput at a lower SNR because the battery problem was significant in WSNs.

## 6. Conclusions

In this paper, the cooperative phase steering (CPS) technique with on-off power control was proposed for generic spectrum sharing-based wireless sensor networks (WSNs), which consisted of a single secondary source node, a single secondary destination node, multiple secondary relay nodes, and multiple primary destination nodes. In the proposed CPS technique, secondary relay nodes that succeeded in decoding the signal from the secondary source node and the transmit power of which was lower than a certain interference threshold were allowed to send the received signal at the second hop so that its interference to the primary destination nodes was maintained lower than an interference constraint. We mathematically analyzed the outage probability of the proposed CPS technique and validated that the proposed CPS technique outperformed the conventional opportunistic relay selection scheme in terms of outage probability and throughput, especially when the number of secondary relay nodes was large.
